# A novel hepatovirus identified in wild woodchuck *Marmota himalayana*

**DOI:** 10.1038/srep22361

**Published:** 2016-02-29

**Authors:** Jie-mei Yu, Li-li Li, Cui-yuan Zhang, Shan Lu, Yuan-yun Ao, Han-chun Gao, Zhi-ping Xie, Guang-cheng Xie, Xiao-man Sun, Li-li Pang, Jian-guo Xu, W. Ian Lipkin, Zhao-Jun Duan

**Affiliations:** 1 National Institute for Viral Disease Control and Prevention, China CDC, Beijing, China; 2 National Institute for Communicable Disease Control and Prevention, China CDC, Beijing, China; 3 Center for Infection and Immunity, Columbia University, New York, NY, USA

## Abstract

Hepatitis A virus (HAV) is a hepatotropic picornavirus that causes acute liver disease worldwide. Here, we report on the identification of a novel hepatovirus tentatively named *Marmota Himalayana* hepatovirus (MHHAV) in wild woodchucks (*Marmota Himalayana)* in China. The genomic and molecular characterization of MHHAV indicated that it is most closely related genetically to HAV. MHHAV has wide tissue distribution but shows tropism for the liver. The virus is morphologically and structurally similar to HAV. The pattern of its codon usage bias is also consistent with that of HAV. Phylogenetic analysis indicated that MHHAV groups with known HAVs but forms an independent branch, and represents a new species in the genus *Hepatovirus* within the family *Picornaviridae*. Antigenic site analysis suggested MHHAV has a new antigenic property to other HAVs. Further evolutionary analysis of MHHAV and primate HAVs led to a most recent common ancestor estimate of 1,000 years ago, while the common ancestor of all HAV-related viruses including *phopivirus* can be traced back to 1800 years ago. The discovery of MHHAV may provide new insights into the origin and evolution of HAV and a model system with which to explore the pathogenesis of HAV infection.

Hepatitis A virus (HAV), a causative agent of water- and food-borne hepatitis, is the only reported member of the genus *Hepatovirus* within the family *Picornaviridae*^1^. HAV is widely distributed in humans and primates and has caused hepatitis A epidemics and outbreaks worldwide, particularly in developing countries^2^,^3^. It has a single-stranded, positive-sense RNA genome of 7,500 base pairs (nt) with a single open reading frame (ORF) flanked by 5′ and 3′ untranslated regions (UTRs). The polyprotein of HAV is cleaved co- and post-translationally into 11 proteins: 4 structural proteins, VP1–VP4 (P1 region) that comprise the viral capsid, and 7 non-structural proteins, 2A–2C (P2 region) and 3A–3D (P3 region)^4^.

The first comparative study of HAV strains in primates demonstrated significant inter-strain heterogeneity^5^. In the mid-1980s, various HAVs were isolated from hepatitis outbreaks of diverse origin^6^,^7^,^8^,^9^. Based on analysis of the 900 nt of the complete VP1 protein, HAV is grouped into six genotypes (I-VI), among which, genotypes I, II and III are of human origin, while IV, V and VI are of simian origin^10^. Despite its considerable genetic variability, HAV exhibits low antigenic variability. Only a single serotype has been described^11^, and the only naturally antigenic variants are HAV strains collected from Old World monkeys^12^. Furthermore, studies using monoclonal antibodies have suggested the presence of a limited number of antigenic epitopes of VP3 and VP1 clustered at the surface of the virus^13^. A recent study using a structure-based predictive method suggested that additional epitopes might exist in VP2 and VP3^14^.

HAV differs greatly from other picornaviruses in many respects^1^, and has specific molecular mechanisms for viral adaptation, including deoptimization of codon usage, Treg shut-off and ‘membrane hijacking’^15^. Furthermore, the clinical spectrum of disease caused by HAV varies considerably, from asymptomatic cases to mild and transient or severe hepatitis^16^. The tropism of HAV for the liver also remains unresolved^17^. Animal models used for HAV most commonly are primates^18^,^19^,^20^,^21^,^22^. No closely related homolog virus has been identified in animals other than primates (human and non-human primates) except for a hepatovirus (*phopivirus*) was very recently discovered in seals^23^. The restricted host range and lack of homologous viruses in other species has hampered the understanding of HAV pathogenesis and immune response. A renewed effort is needed to better characterize animal HAV-related viruses and to develop new animal models for hepatitis A virus infection^24^.

Here, we report on the discovery of a novel *Hepatovirus* from wild woodchuck *Marmota Himalayana*, provisionally named *Marmota Himalayana* hepatovirus (MHHAV) which may represent a new species and serotype in the genus *Hepatovirus* within the family *Picornaviridae.*

## Results

### MiSeq high-throughput sequencing

Two unique reads with lengths of 2,738 nt (contig1) and 3,324 nt (contig2) were assembled from the initial sequencing data. Blastn showed that contig1 had the best hit to simian HAV, with 75% nt identity, while contig2 had the best hit to human HAV, with 70% nt identity. Contigs1 and 2 were confirmed using specific primers.

### Genomic characterization

Based on the sequences of contigs1 and 2, specific primers were designed to generate overlapping polymerase chain reaction (PCR) products. Two full-length genome sequences showing 99% nt identity from two different samples (ID 2 and 3) were acquired and deposited into GenBank under accession numbers KT229611 and KT229612. The MHHAV genome comprises 7,566 bp (excluding the polyadenylated tail), with a 713 nt 5′ UTR, an ORF of 6,756 nt (encoding a potential polyprotein precursor of 2,252 amino acids [aa]), followed by a 100 nt 3′ UTR and a poly (A) tail. The base content of the MHHAV genome is 31.39% A, 13.30% C, 20.41% G, and 34.89% U, with a pyrimidine content of 48.19%, which is similar to the known HAVs (simian HAV prototype, 49.21%; human HAV prototype, 48.95%; *phopivirus* prototype, 46.83%). Sequence analysis revealed that the polyprotein of MHHAV showed 67% aa identity to that of HAV and 58% aa identity to that of *phopivirus*. Furthermore, MHHAV displays a strong codon bias that is complementary to that of the woodchuck (Table 1). The rare codons of MHHAV are very abundant in woodchuck codons (Table 2).

**Figure 1:**
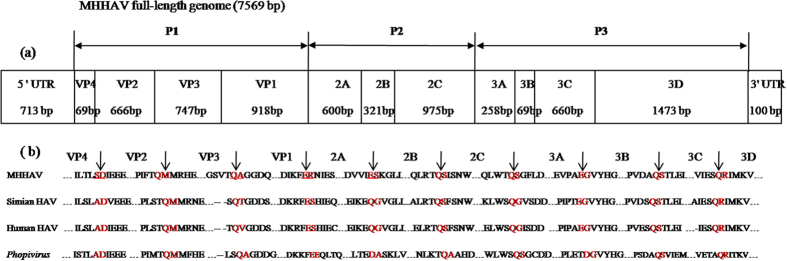
MHHAV genome organization and cleavage sites. (**a**). Structure map of the MHHAV genome. P1, encoding the viral structural proteins VP4-VP2-VP3-VP1; P2 and P3 are nonstructural proteins, of which, P2 contains the 2A–2C regions and P3 the 3A–3D regions. (**b**). Amino acid sequences of MHHAV, the prototypic human and simian HAVs and phopivirus adjacent to the predicted protease cleavage sites (10 aa on each side are shown). The amino acids in red indicated by an arrow represent cleavage sites.

A hypothetical cleavage map of the MHHAV polyprotein was derived by alignment with other HAVs (Fig. 1a). Only 3 cleavage sites are conserved in MHHAV, primate HAV and *phopivirus* (VP2–VP3, 3B–3C, 3C–3D) (Fig. 1b). The P1 region of MHHAV is 2,400 nt in length, 27, 15 and 90 nt longer than those of the prototypic human HAV, simian HAV and *phopivirus*, respectively. Interestingly, compared to previously known HAVs an additional 18 nt sequence was detected in the VP1 region (positions 3,009–3,026 nt, sequence: TCTTCCTCTAGGAGAACA, coding for six aa “SSSRRT”); whereas only 15 nt was found in the recently reported *phopivirus* (TCCTCTAGGAGAACA). An RGD motif was found in the middle region of VP3 in MHHAV.

The P2 regions of the prototypic human and simian HAVs are 1,893 nt in length, but it is 1,896 nt in MHHAV and 1,932 nt in *phopivirus*; highly conserved aa motifs, GXXGXGKT (G_1250_KRGGGKS) and D_1301_DIGQ, in the nt-binding domain of the putative picornavirus NTPase and helicase, were also found in the 2C protein of MHHAV. The P3 region of MHHAV is 2,460 nt in length, which is 48 and 51 nt longer than those of the prototypic human and simian HAV, respectively, but 15 nt shorter than that of the *phopivirus*. The 3D region includes the KDELR, YGDD and FLKR motifs of the RNA-dependent RNA polymerase.

### Antigenic site analysis

HAV has a conformation-dependent immunodominant neutralization site. Residues S102, V171, A176, and K221 of VP1 as well as Q70, S71, E74 and 102–121 of VP3 have been implicated in neutralizing epitopes; residues 71 and 198 of VP2 as well as residues 89–96 of VP3 may harbor other epitopes^14^. Sequence alignment showed that all the aa in the antigenic sites were different between MHHAV and HAV prototype Fig. S1a) with the exception of S102 of VP1 and T71 of VP2. In addition, only two aa (A70 of VP3 and T71 of VP2) were identical between MHHAV and simian prototype. Furthermore, three aa are the same between MHHAV and the recently reported *phopivirus*, T74 of VP3 as well as T71 and P198 of VP2. The detailed alignment of the MHHAV capsid protein with counterparts was shown in Fig. S2. The antigenic sites in the MHHAV model are presented in both cartoon (Fig. S1a) and surface (Fig. S1c) forms.

### Secondary RNA structure of the 5′ UTR

**Figure 2:**
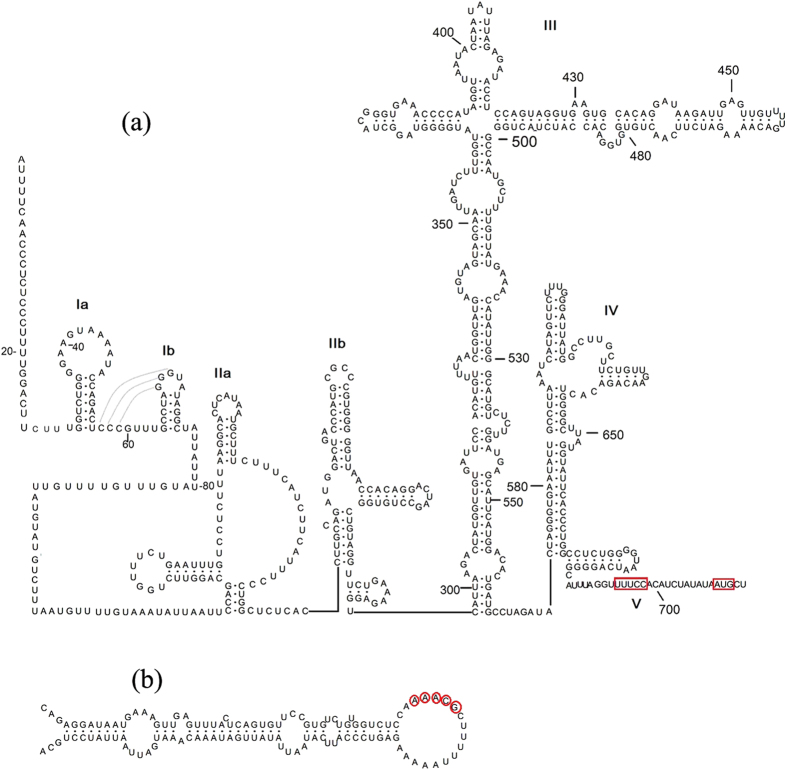
Predicted partial secondary structure of MHHAV. (**a**) Secondary structure of the MHHAV 5′ UTR; domains are labeled I to V. The putative initiator codon (AUG) and UUUCC sequence are indicated in red. (**b**) Cis-acting secondary structures of MHHAV in the 3D *pol*-coding region.

The 5′ UTR of MHHAV is 20 nt shorter than that of human HAV (734 nt), 45 nt and 73 nt longer than those of simian HAV (669 nt) and *phopivirus* (648 nt), respectively. It shares 56.72%, 54.48% and 41.73% nucleotide identity with human HAV, simian HAV and *phopivirus*, respectively. The predicted secondary structure of MHHAV (Fig. 2a) shows that MHHAV 5′ UTR contains five major structural domains (including six stem-loops) labeled from I to V beginning at the 5′ terminus of the 5′ UTR, and lacks the first domain found in human HAV. The first domain (stem-loop Ia and Ib) of MHHAV corresponds to domain II (stem-loop) of human HAV. The predicted secondary structure of simian HAV 5′ UTR (Fig. S3) is similar to that of human HAV but lacks the first domain and stem-loop Ia. The stem-loop Ia and Ib of domain I, present in human HAV domain II, may form a pseudoknot; however, the pseudoknot in domain V of human and simian HAVs was not found in MHHAV.

The segment from 79 to 120 nt of the MHHAV 5′ UTR represents a polypyrimidine tract that has been suggested not to be involved in the formation of conserved helical structures^25^. In the 5′ UTR secondary structure of MHHAV, stem-loop III is a long multi-loop cloverleaf structure that corresponds in position and shape to the previously designated stem-loop IV (in primate HAVs and *phopivirus*), is an internal ribosome entry site (IRES). The IRESes in MHHAV, primate HAV and *phopivirus* have highly conserved base paired regions governing internal translation initiation and belong to IRESes of type III.

Two important motifs near the 3′ border of the picornavirus IRES were also found in the MHHAV 5′ UTR. The first motif is an UUUCC sequence (box A) within the second pyrimidine-rich tract; the second motif is an AUG triplet (box B) that functions as an initiation codon in HAV and *phopivirus*^26^. Similar to human and simian HAVs a putative cis-acting RNA replication element (*cre*) is located near the 5′ end of the 3D Pol-coding sequence of MHHAV. It contains a top loop of 18 nt with a stem segment of 34 nt, interrupted by four internal loops and two 1 nt bulges (Fig. 2b). However, in spite of the 68% nt identity between the *cre*s of MHHAV and *phopivirus*, the similar region in *phopivirus* includes three loops and a stem segment (Fig. S4).

### Detection of MHHAV in wild woodchucks

**Figure 3:**
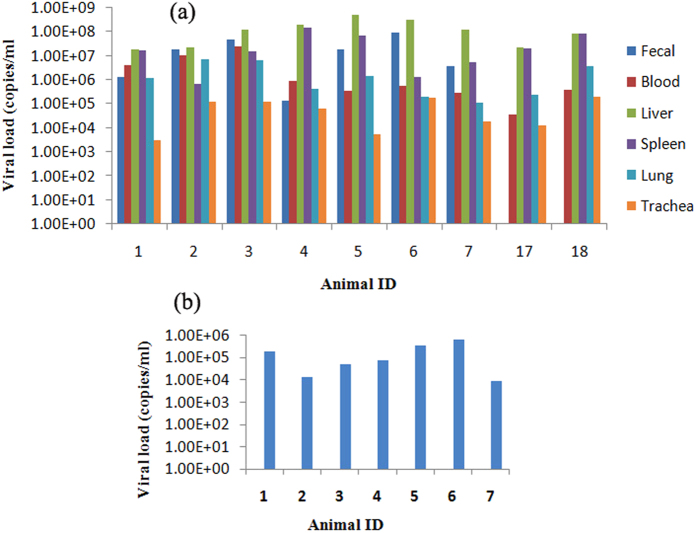
Positive-strand and negative-strand viral RNA copy number determined by real-time PCR. (**a**) Numbers of positive-strand viral RNA copies in feces, blood and other tissues. The highest viral RNA load was in the liver, and the lowest in the trachea. *T*-test showed the viral loads in liver and other tissues were statistically significant (blood, p = 0.000; spleen, p = 0.022; lung, p = 0.000; trachea, p = 0.000). (**b**) Negative-strand RNA in the liver. The amplification curves of the negative-strand viral RNA were not observed in all collected tissues except for the liver. Note: “ID” stands for “identifier”.

Sixteen (16.16%) (ID1-16) of 99 enteric lysates from wild woodchucks were positive for MHHAV RNA by RT-PCR, among which seven animals (ID1-7) were chosen for the collection of blood, liver, spleen, lung, and trachea samples. All sample types were MHHAV RNA-positive, with the highest virus load in the liver and the lowest in the trachea. *T*-test showed the viral loads in liver and other tissues were statistically significant (blood, p = 0.000; spleen, p = 0.022; lung, p = 0.000; trachea, p = 0.000). Two woodchucks (ID 17 and 18) had MHHAV RNA in their tissues and blood, but not in their feces (Fig. 3a). The sequences of the complete VP1 region amplified from these positive samples were determined. The 34 VP1 sequences showed 99% nt identity. Sequences within an individual woodchuck showed 100% nucleotide identity. All sequences were deposited into GenBank under accession numbers KT229577-KT229610. Furthermore, negative-stranded MHHAV RNA was detected only in the seven MHHAV-positive livers, while it is not detected in the spleen, lung, trachea, blood, and feces samples (Fig. 3b).

### Phylogenetic and evolutionary analysis

**Figure 4:**
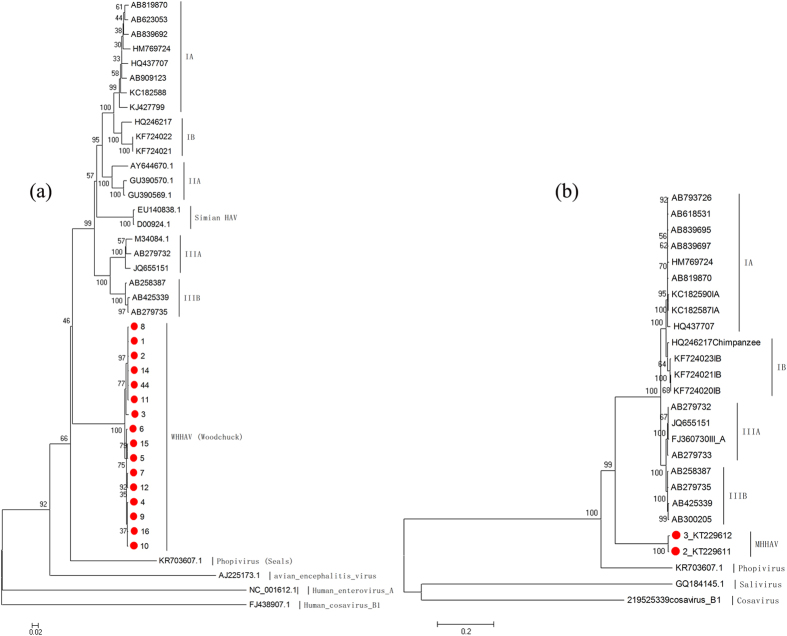
Phylogenetic analysis of sequences of MHHAV: (**a**) VP1 region (nucleotide); (**b**) polyprotein (amino acid). The tree was constructed using the neighbor-joining method by MEGA ver. 5 with 1,000 bootstrap replicates. The virus in this study is indicated by the red “•.” Bootstrap values are shown on the branches. Results showed that MHHAV formed a distinct lineage to the known primate HAVs and phopivirus.

Recombination analysis showed no evidence of inter-genotype recombination among primate HAVs, *phopivirus* and MHHAV (Fig. S5). Phylogenetic analysis suggested that MHHAV forms a distinct lineage compared to previous reported HAVs by the neighbor-joining method and 1,000 bootstrap replications. In contrast, the distance between *phopivirus* and previously reported HAVs is much closer (Fig. 4).

**Figure 5:**
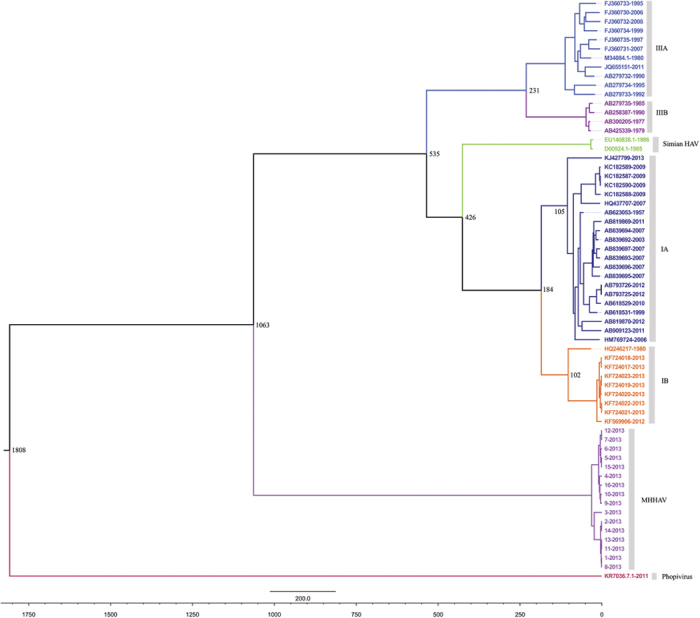
The Bayesian Markov Chain Monte Carlo (MCMC) tree of the VP1 regions of MHHAV and the known primate HAVs and phopivirus. Horizontal branches are drawn to scale of estimated year of divergence, with tip times reflecting sampling data (year). The estimated time for the most recent common ancestors of the major nodes of the lineages are shown. IA, IB, IIIB: subgenotypes of HAV genotypes I and III.

Under the best-fit model, the mean substitution rate was 8.62 × 10^−4^ substitutions per site per year (ssy), with a 95% HPD of 6.96 × 10^−3^–7.03 × 10^−3^. The time of the most recent common ancestor of MHHAV and the primate HAV isolates was estimated to be around 1,000 years ago. In contrast, the common ancestor of MHHAV and *phopivirus* was estimated to be 1800 years ago (Fig. 5).

### Electron microscopy

**Figure 6:**
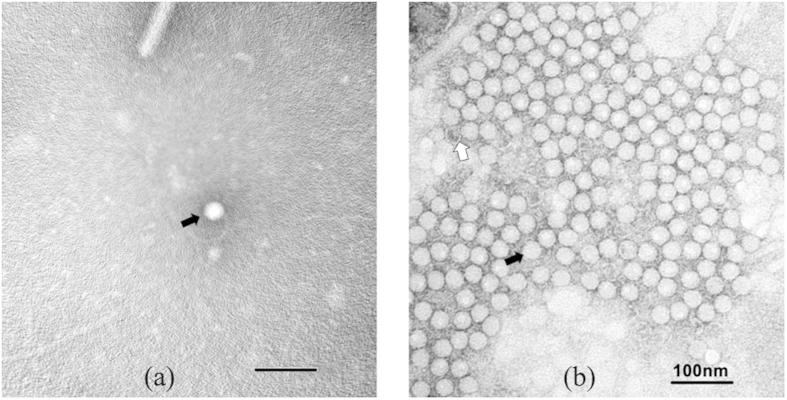
Spherical, non-enveloped virus particles of ~27 nm in diameter visualized by negative-staining electron microscopy: (**a**) unclumped MHHAV; (**b**) immune-complexed MHHAV. The black arrow indicates an intact particle; the white arrow indicates a potential empty particle.

MHHAV was visualized by negative-staining electron microscopy, which revealed the presence of spherical, non-enveloped virus particles of ~27 nm in diameter, morphologically similar to HAV. Chloroform-purified virions were only rarely detected in feces, but were readily apparent following incubation with a polyclonal antibody (Fig. 6).

## Discussion

HAV continues to be a source of morbidity and mortality despite the availability of an effective vaccine^15^,^27^. Recently a new HAV-related hepatovirus known as *phopivirus* was reported in seals^23^. Here, we report on the identification of a novel *hepatovirus*, tentatively named MHHAV, from wild woodchuck *Marmota Himalayana*. The purified virus particles from the fecal sample are morphologically similar to HAV virions by negative-staining electron microscopy. The complete genome and VP1 capsid protein of MHHAV was best hit to simian HAV, with 67% nt and 75% aa identities, respectively. Based on the classification of enteroviruses, in which a VP1 sequence identity of 70 to 85% is defined as a heterologous serotype, it is suggested that MHHAV may be a new serotype of HAV. Phylogenetic analyses of the polyprotein and VP1 protein indicate that MHHAV forms a separate branch from previous HAVs and may represent a new species in the genus *Hepatovirus* within the family *Picornaviridae*.

Although MHHAV has a genome organization identical to that of previously reported HAVs, the length of MHHAV polyprotein is different to those of the prototypic human HAV, simian HAV and *phopivirus*, respectively. Furthermore, the aa sequences of the predicted cleavage sites of MHHAV differ substantially from these viruses with only three cleavage sites (VP2/VP3, 3B/3C, and 3C/3D) conserved. The common motifs in the non-structural proteins of picornaviruses, such as the NTPase and helicase motifs, were also found in MHHAV. In most picornaviruses, RGD motif is located near the C terminus of VP1^28^, however, this motif was found in the middle of the VP3 region in MHHAV, primate HAVs and *phopivirus*. The RGD motif is conserved in picornaviruses and functions in recognizing and attaching to host cells or enabling cell-to-cell and cell-to-matrix interactions^29^,^30^. It is inferred from differences in the positions of RGD motifs that HAV interactions with host cell-surface integrins differ from those of other picornaviruses^14^. Furthermore, hepatovirus differ from other picornaviruses in that they rarely use codons most often preferred by their hosts^23^,^31^. MHHAV appears to follow HAV and *phopivirus* in codon usage. We speculate that this strategy may minimize direct competition of hepatovirus with host cell systems and enable persistence^31^.

The antigenic sites of HAVs have been characterized by several research teams^13^,^14^,^32^. Sequence alignment showed that these antigenic sites differed between MHHAV, primate HAVs and *phopivirus*. Further sequence alignment of the whole genome of MHHAV, primate HAVs and *phopivirus* revealed an 18 nt and 15nt insertion encoding six aa ([S]SSRRT) at the C terminus of VP1, with three or two potential *O*-glycosylation sites (glycosylation site prediction website: http://www.cbs.dtu.dk/services/NetNGlyc/). As glycosylation is essential for antigen processing and presentation and VP1 is a major antigenic protein, we speculate that these sequence differences may indicate antigenic differences between MHHAV and other HAVs. However, the real antigenic characteristics should be determined by a neutralization assay when an *in vitro* cell culture system for these viruses has been developed.

The predicted secondary structure of the 5′ UTRs of MHHAV, human HAV,, simian HAV and *phopivirus* is very similar. The most important structure in the 5′ UTR is the IRES, which directs internal initiation of translation^25^. The IRESes of primate HAVs, MHHAV and *phopivirus* exhibit evolutionarily conserved secondary structure including a long multi-loop cloverleaf structure^23^, which has been grouped into type III. This is different from IRESes of *poliovirus* and human *rhinovirus* (type I), *encephalomyocarditis virus* and foot-and-mouth disease virus (type II) and HCV like IRES (type IV)^33^,^34^. A conserved RNA structure, the 110 nt HAV *cre* element located near the 5′ end of the 3D^Pol^ region, is present in both human and simian HAVs as well as in the distantly related *avian encephalomyelitis virus*^35^, but the similar *cre* element was not present in the corresponding region in phopivirus. However, this element was also found in MHHAV, as was the AAACA/G motif that serves as the template for uridylylation of VPg by a slide-back mechanism^35^. These findings suggest that MHHAV might have replication mechanisms and tissue tropism similar to those of other known HAVs, but the shape and position of *cre* element in *phopivirus* were still unknown. Further studies are needed to address the subtle difference in the secondary structure of the 5′ UTR among MHHAV, HAV and *phopivirus*.

HAV is highly transmissible and HAV infection is acquired primarily by the fecal-oral route. The poor sanitary condition can cause HAV outbreak locally. In the present study, 16 out of 99 (16.16%) wild woodchucks were detected to carry the MHHAV. Furthermore 34 MHHAV VP1 sequences from different animals in this study shared 99% nt identity and sequences in the same woodchuck showed 100% nt identity. All these indicated that an MHHAV outbreak might happen in wild woodchucks at the time we collected the samples. As anticipated for an HAV replication in the liver^36^, negative-sense RNA complementary to the positive-sense genomic MHHAV RNA was only detected in the liver, however, though with the highest RNA viral load in the livers, MHHAV distributed widely in different organs in the wild woodchucks, which was in accordance with the previous studies that HAV (*phopivirus* included) can also be detected in extrahepatic organs in the hosts^22^,^23^,^37^,^38^. This phenomenon may be partly explained by the fact that the HAV cellular receptor 1 was expressed wildly in different organ, such as liver, spleen, kidney and testis^39^, so these organs can capture HAV virons. However, they cannot support the virus replication by some specific reasons. These findings suggest that the liver is the target organ of MHHAV infection and replication in wild woodchucks. Although its hepatotropism and ability to cause disease remain to be determined, the presence of MHHAV in the liver of wild woodchuck *Marmota Himalayana* is reminiscent of human HAV infection.

The *phopivirus* reported in seals in USA^23^ has similar geomic organization, codon usage bias and hepatic tropism to HAV and MHHAV. Phylogenetic analyses in this study indicated that the *phopivirus* is more distant to the previously reported HAVs than MHHAV. Evolutionary analysis suggested that *phopivirus* has a common evolutionary ancestry with HAVs and MHHAV. The estimated substitution rate of these viruses was 8.62 × 10^−4^ ssy, similar to a French study based on VP1 sequences from primate genotype IA HAVs (9.76 × 10^−4^ ssy)^40^. This substitution rate is lower than those found in other picornaviruses^41^,^42^. However, the common ancestor of MHHAV, primate HAVs and *phopivirus* was older than that of MHHAV and primate HAVs, indicating that the diversity and evolutionary pathway of HAV are far more complex than previously thought.

Human hepatotropic viruses or related viruses that infect wild woodchuck include *woodchuck hepatitis virus* (WHV) and *hepatitis delta virus* (HDV)^43^,^44^. Natural infection with WHV results in liver disease similar to that induced by HBV in humans^45^. Woodchuck *Marmota monax* is commonly used as an animal model for *hepatitis B virus* (HBV) infection^46^,^47^. There are currently no non-primate models of HAV infection. Guinea pigs can be infected by HAV but do not develop signs of disease or seroconvert^38^. The discovery of MHHAV in woodchuck may facilitate the development of a new tractable animal model of human HAV infection and thus provide further insights into the evolution and pathogenesis of, HAVs.

## Materials and Methods

### Specimens and high-throughput sequencing

Ninety-nine wild woodchucks were caught from Haixi, Qinghai Province in 2013. Enteric lysates of all the animals and the liver, spleen, lung, and trachea specimens of some animals were collected after exsanguination. The samples were transported on dry ice and stored at −80 °C at the China Center for Disease Control (CDC). After dilution (1:5 ratio, wt/vol) and filtration (0.45 μm and 0.22 μm membranes), total nucleic acid was extracted from 99 enteric lysates, followed by cDNA synthesis. Random PCR amplification was performed on each sample using primers with different barcodes. The PCR products were pooled for sequencing on the Illumina MiSeq platform (Illumina, San Diego. CA). Metagenomic profiling of the shotgun datasets was carried out using the customized informatics pipeline VirusSeeker to computationally identify viral sequences^48^. The study protocol was approved by the Ethics Committee of the China CDC, and was performed according to Chinese ethics laws and regulations. Furthermore, the methods were carried out in accordance with the approved guidelines.

### Full-length genomic amplification

To determine the full-length genomic sequence of MHHAV, primers were initially designed based on contigs obtained by miseq high-throughput sequencing. Further synthesis was based on newly amplified MHHAV sequences. Long fragments (1500–3000 bp in length) were amplified for final confirmation. All PCR amplifications were performed using ExTaq DNA polymerase. The extreme 5′ and 3′ ends of the genome were determined using a SMART RACE cDNA Amplification Kit (Clontech, US) and Genome Walking Kit (Takara, Japan). Sequences were assembled and manually edited to produce the final sequence of the viral genome. Codon usage was assessed both for the MHHAV and woodchuck. For woodchuck, codon usage tables were obtained from a database based on genomes in NCBI GenBank (http://www.kazusa.or.jp). For MHHAV, codon usage frequencies were determined by the Cusp program (http://emboss.sourceforge.net/apps/cvs/emboss/apps/cusp.html).

### Detection of MHHAV and amplification of complete VP1 region

The presence of the MHHAV in wild woodchucks was confirmed by using the primers (forward: 5′-GATCCACAATATCCAGTTTGGG-3′, reverse: 5′- CATGGTGTGCTACATTACTAGG-3′) targeting 658nt region spanning the VP2/VP3-coding junction of the virus using RT-PCR. Complete VP1 genome sequences were amplified with the primers listed below: forward: 5′-CTTTGAAGCAGGCAACTACTGGACC-3′, reverse: 5′- AAGAGATAGGTTCCCCTGCTTGTGT-3′.The reaction mixture included 20 pmol of each primer and 2.5 U of ExTaq DNA polymerase (Takara Bio). After 5 min at 94 °C, 35 cycles of amplification (94 °C for 30 s, 59 °C for 30 s, and 72 °C for 1 min) were performed, followed by a 7-min extension at 72 °C. Products were resolved on a 1.5% agarose gel and purified (QIAquick PCR purification kit, QIAGEN). Nucleotide sequences were determined using the Big-Dye terminator cycle sequencing kit and the ABI Prism 310 Genetic Analyzer (Applied Biosystems Inc.). Sequences were determined and analyzed using the software package DNAStar.

### Quantitative PCR

Viral RNA copies of MHHAV were quantified by real-time PCR. A forward primer (MHHAVF: 5′- GTCCTCTTTAAGGCACTCAT -3′), a reverse primer (MHHAVR:5′-TGGGTCAGTCCATCTGGCAAG-3′), and a probe (MHHAV Probe: 5′-FAM- CATCTTCATTTCCCTGGCTCTCACC-MGB -3′) were designed from sequences in the 5′ UTR region of the virus. The reaction condition involved 50 °C for 30 min, 95 °C for 10 min, and 40 cycles of 95 °C for 15 s and 58 °C for 30 s.

### Negative-strand MHHAV RNA testing

A tagged primer system was used to detect the negative-strand RNA. Negative-strand cDNA was generated with Tag MHHAV primer (5′ - CCTCCGCTGCCATCTGATTGCGTCCTCTTTAAGGCACTCAT - 3′, targeting 5′ UTR of MHHAV), performed at 65 °C for 5 min, 25 °C 10 min, 50 °C 50 min and 72 °C 15 min. Then quantitative PCR was established to quantify the negative-strand RNA, a forward primer (TagF: 5′- CCTCCCGATCATCTGGTTGC -3′), a reverse primer (UTRR:5′-TGGGTCAGTCCATCTGGCAAG -3′), and a probe (NProbe: 5′- FAM-CATCTTCATTTCCCTGGCTCTCACC-MGB -3′) were used. The reaction condition involved 50 °C for 2 min, 95 °C for 10 min, and 40 cycles of 95 °C for 15 s and 58 °C for 30 s.

### Recombination and phylogenetic analysis

Complete genomes of the known HAVs were downloaded. To detect potential combination, aligned sequences were analyzed by using the Boots canning method and the neighbor-joining algorithm was run with 100 pseudo replicates implemented in Simplot software. Phylogenetic tree were performed using nucleic acid sequences of VP1 and polyprotein by the Neighbor-joining method and subsequently subjected to bootstrap analysis with 1000 replicates. Tree figures were produced using MEGA software (version 5).

### Evolutionary analysis

To precisely estimate MHHAV, phopivirus and HAV substitution, a Bayesian Markov chain Monte Carlo (MCMC) approach was implemented in the BEAST package (v 1.8.2, available from http://beast.bio.ed.ac.uk/downloads). The jModelTest software 2.1.7 was used to identify the optimal evolutionary, Akaike Information Criterion and hierarchical likelihood ratio test suggested that the GTR (general time reversible) + Γ (gamma distributed rate variation) model best fitted the sequences in this study. Different population dynamic models were used (constant size, exponential growth, logistic growth, expansion growth and Bayesian skyline). The MCMC analysis was performed with 50 million generations and sampled every 1000 generations with 10% burnin. The results were computed and analyzed using Tracer 1.6. The effective sample size values for the estimated parameters in the MCMC analysis were greater than 200. Statistical uncertainty in the data was reflected in the 95% highest probability density values (HPD).

### RNA structure prediction of the 5′ UTR

The secondary structure of the 5′ UTR RNA of MHHAV was predicted using consecutive fragments of the complete nucleotide sequence of the 5′ UTR of MHHAV and a thermodynamic folding energy minimization algorithm with RNA structure software (version 5.3); the graph was integrated using RnaViz (version 2.0.3).

### Structure prediction of MHHAV

Based on a previously solved HAV particle structure (PDB: 4QPG)^14^, the structure of MHHAV was modeled using Phyre^49^ (http://www.sbg.bio.ic.ac.uk/phyre2/html/page.cgi?id=index).

### Virus purification

Stool samples were diluted to 20% suspensions in phosphate-buffered saline (PBS). Beads and chloroform were added to the suspensions, followed by 20 min centrifugation at 1,500 × g. Supernatant was collected and then subjected to a single ultracentrifugation step through a discontinuous sucrose/glycerol density gradient for HAV purification, as described previously^28^.

### Electron microscopy

Fifty-microliter volumes of chloroform-purified MHHAV (1 × 10^7^ copies/ml) in PBS were examined directly by negative staining with 1% phosphotungstic acid (pH 6.8). Chloroform-purified MHHAV (450 μl) was incubated with a 1:10 dilution of 50 μl MHHAV polyclonal antibody (BALB/c mice immunized subcutaneously with purified MHHAV) at 37 °C for 1 h. Following centrifugation at 23,000 rpm for 1 h, the sediment was resuspended in 50 μl PBS and the suspension subjected to negative staining. Grids were examined using a transmission electron microscope (TECNAI 12, FEI, Blackwood, NJ).

### Statistical Analysis

The statistical significance of viral load means between the liver and other tissues was assessed using the Student’s t test, and the statistical analyses were performed using SPSS 16.0.

## Additional Information

**How to cite this article**: Yu, J.- *et al*. A novel hepatovirus identified in wild woodchuck *Marmota himalayana. Sci. Rep.* **6**, 22361; doi: 10.1038/srep22361 (2016).

## Supplementary Material

Supplementary Information

## Figures and Tables

**Table 1:** Relative occurrence of synmonymous codons for each amino acid.

Amino acid (symbols)	Codon (s)	Occurrence (%) of codon in virus/host			
		MHHAV	Woodchuck	Human	HAV
Phenylalanine (Phe, F)	UUU	100.00	48.83	86.70	100.00
	UUC	13.33	100.00	100.00	28.20
Leucine (Leu, L)	UUA	91.57	9.57	19.40	49.32
	UUG	100.00	25.18	32.60	100.00
	CUU	20.48	20.29	33.30	44.66
	CUC	1.20	53.90	49.50	8.22
	CUA	4.82	14.46	18.20	9.32
	CUG	10.84	100.00	100.00	26.03
Isoleucine (Ile, I)	AUU	100.00	56.46	76.90	100.00
	AUC	5.45	100.00	100.00	16.63
	AUA	46.36	30.03	36.10	31.62
Valine (Val, V)	GUU	100.00	24.96	39.10	100.00
	GUC	12.36	58.23	51.60	14.81
	GUA	24.72	14.43	25.30	16.99
	GUG	35.96	100.00	100.00	45.15
Serine (Ser, S)	UCU	100.00	86.18	77.90	100.00
	UCC	16.67	90.24	90.80	29.87
	UCA	79.17	42.55	62.60	88.93
	AGU	40.28	45.53	62.10	33.56
	AGC	5.56	100.00	100.00	6.71
	UCG	1.39	18.16	22.60	4.70
Proline (Pro, P)	CCU	100.00	91.58	88.40	100.00
	CCC	9.62	100.00	100.00	21.74
	CCA	71.15	53.16	85.40	81.64
	CCG	3.85	23.95	34.80	2.42
Threonine (Thr, T)	ACU	100.00	55.51	69.30	100.00
	ACC	9.21	100.00	100.00	20.74
	ACA	65.79	38.67	79.90	92.59
	ACG	1.32	17.88	32.30	4.44
Alanine (Ala, A)	GCU	100.00	85.96	66.40	100.00
	GCC	12.68	100.00	100.00	28.37
	GCA	50.70	40.99	57.00	58.51
	GCG	1.41	26.76	26.70	1.42
Tyrosine (Tyr, Y)	UAU	100.00	55.35	79.70	100.00
	UAC	12.99	100.00	100.00	28.41
Histidine (His, H)	CAU	100.00	61.04	72.20	100.00
	CAC	20.59	100.00	100.00	22.11
Glutamine (Gln,Q)	CAA	100.00	26.62	36.00	92.86
	CAG	53.57	100.00	100.00	100.00
Asparagine (Asn, N)	AAU	100.00	75.00	89.00	100.00
	AAC	9.09	100.00	100.00	21.75
Lysine (Lys, K)	AAA	100.00	74.48	76.50	100.00
	AAG	43.16	100.00	100.00	58.85
Asparitic acid (Asp, D)	GAU	100.00	58.87	86.90	100.00
	GAC	12.40	100.00	100.00	21.04
Glutamic acid (Glu, E)	GAA	100.00	56.37	73.20	100.00
	GAG	38.10	100.00	100.00	76.51
Cysteine (Cys, C)	UGU	100.00	61.04	84.10	100.00
	UGC	13.51	100.00	100.00	26.58
TryPtophan (Trp, W)	UGG	100.00	100.00	100.00	100.00
Arginine (Arg, R)	CGU	1.32	27.60	36.90	4.21
	CGC	0.00	44.80	85.20	2.11
	CGA	2.63	28.32	50.80	3.51
	CGG	2.63	45.88	93.40	2.11
	AGA	100.00	91.04	100.00	100.00
	AGG	21.05	100.00	98.40	31.58
Glycine (Gly, G)	GGU	100.00	44.64	48.60	60.87
	GGC	10.17	100.00	100.00	27.90
	GGA	100.00	74.40	74.30	100.00
	GGG	27.12	84.82	69.80	34.78

Note: The codon of highest frequency for each amino acid was assigned to 100%, the occurrence of other codons for each amino acid was relative to the occurrence of the most abundant frequency.

**Table 2:** Rare codon usage in hosts and viruses.

Source	RCs number	RCs abundant (100%) in host	No. of Amino acids with RCs
Woodchuck	13	NA	8
MHHAV	29	47	15
Human	5	NA	4
HAV	25	12	15
poliovirus	7	0	4
Seal	9	NA	6
phopivirus	20	9	12
