# Supplementary material for: A novel hepatovirus identified in wild woodchuck Marmota himalayana
Source: Sci Rep. 2016 Feb 29;6:22361. doi: 10.1038/srep22361 (PMC4770319; doi:10.1038/srep22361)
Supplement: Supplementary Information [file srep22361-s1.doc]

**A novel hepatovirus identified in wild woodchuck *Marmota himalayana***

Jie-mei Yu^1⊥^, Li-li Li^1⊥^, Cui-yuan Zhang^1^, Shan Lu^2^, Yuan-yun Ao^1^, Han-chun Gao^1^, Zhi-ping Xie^1^, Guang-cheng Xie^1^, Xiao-man Sun^1^, Li-li Pang^1^, Jian-guo Xu^2^, W. Ian Lipkin^3^, Zhao-Jun Duan^1*^

**Supporting Information**


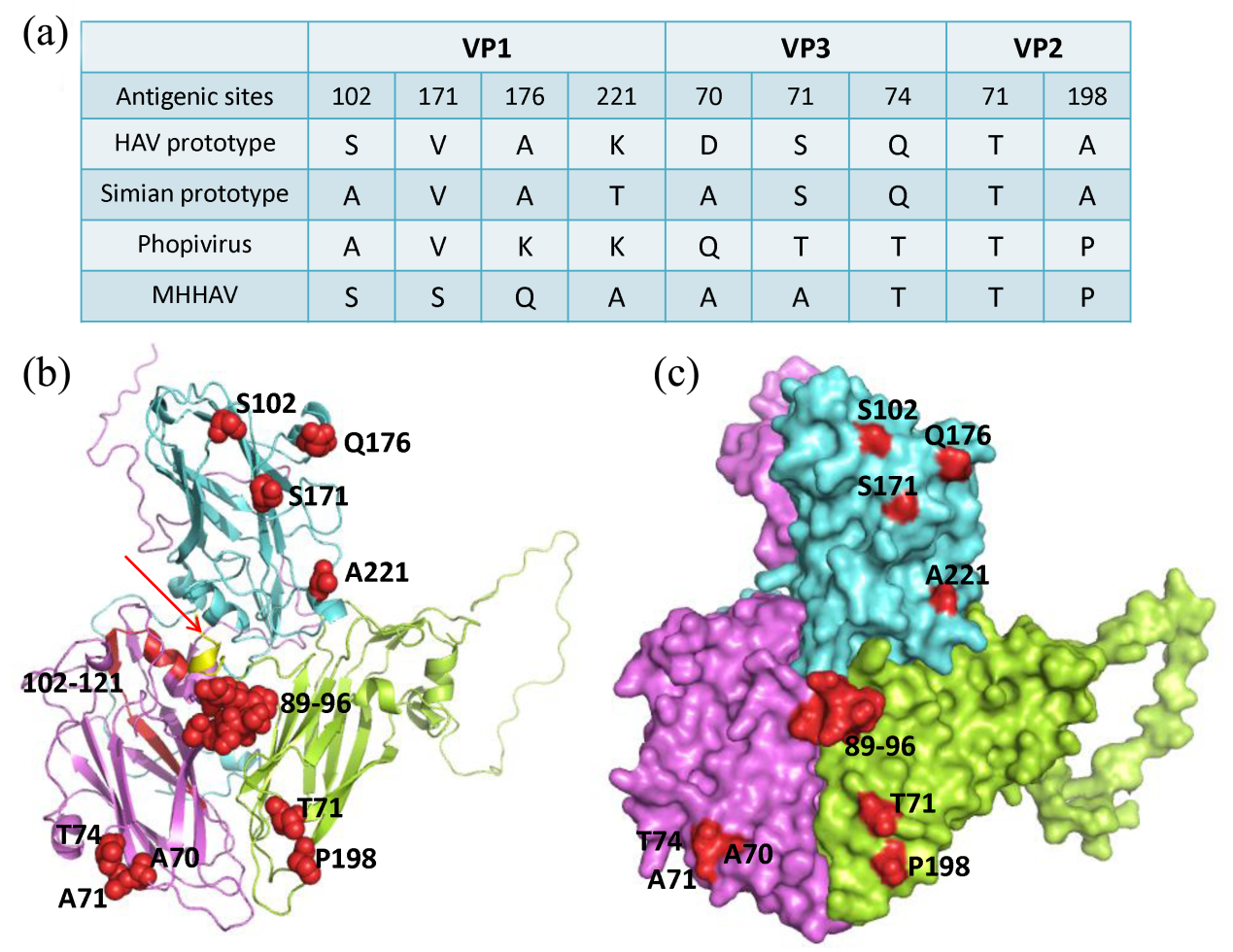


Figure S1. Antigenic site analysis. A. The sequence alignment of the amino acids of the major antigenic site reported previously. B. Cartoon of the MHHAV model structure. The model was constructed using Phyre and analysized with Pymol. Aquamarine, VP1; violet, VP3; green, VP2. Antigenic sites are indicated by red spheres. The VP1 carboxyl terminus is shown in yellow and indicated by the red arrow. C. Surface view of the MHHAV model. Antigenic sites are shown in red.


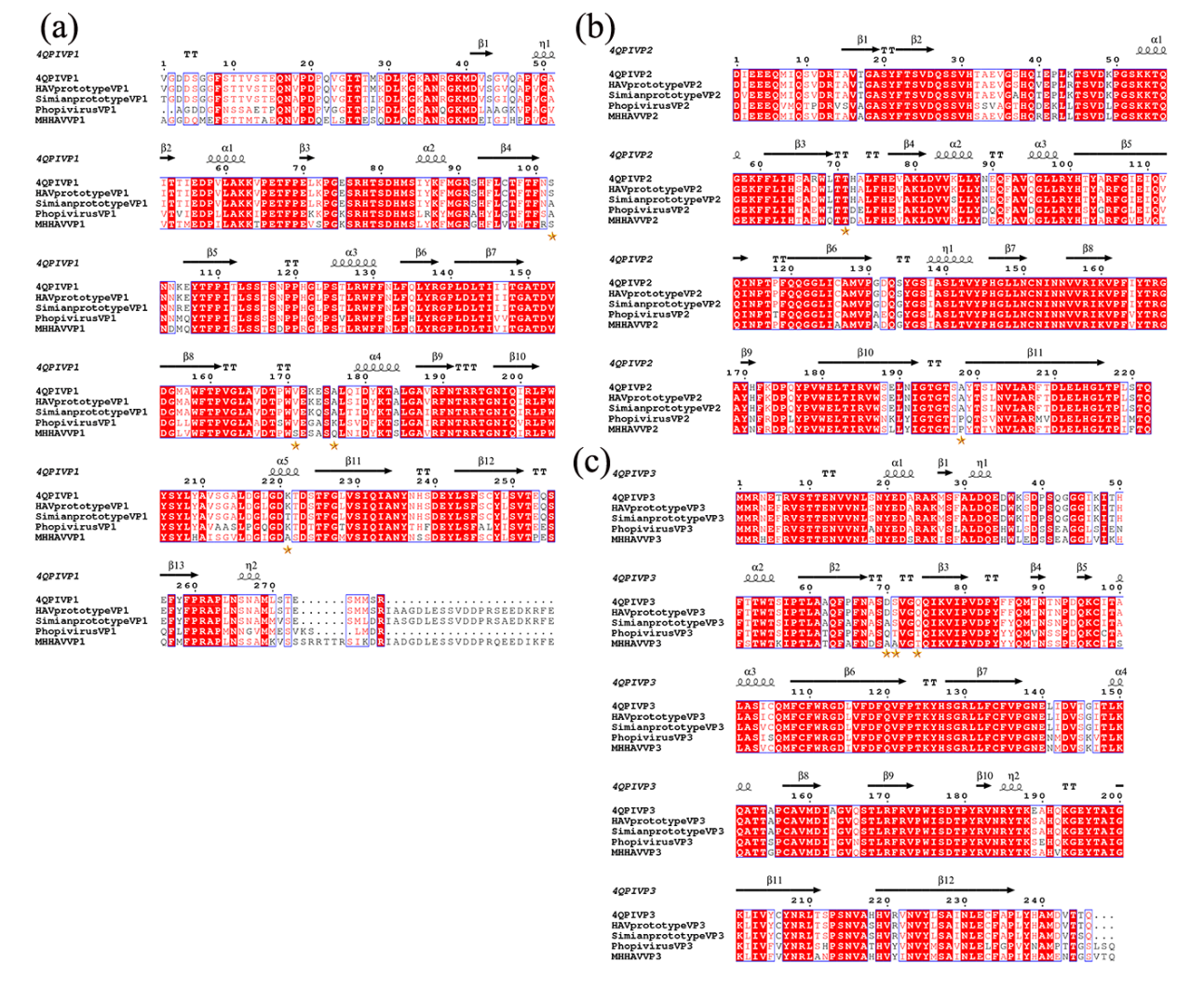


Figure S2. Alignment of antigenic sites in the capsid proteins (VP1, VP2 and VP3) of MHHAV, the prototypic human and simian HAVs and phopivirus. Amino acids marked with asterisks are previously reported antigenic sites.


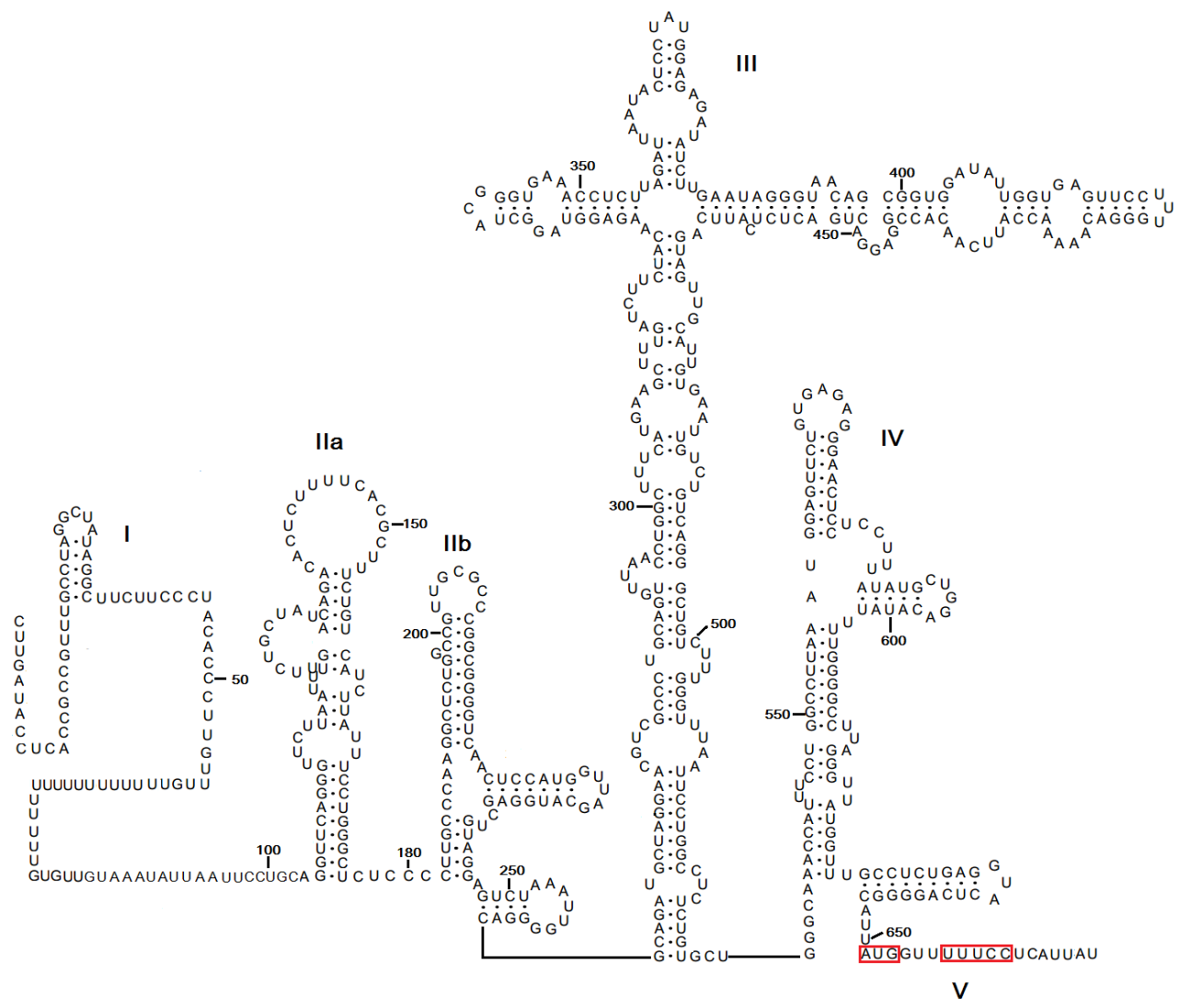


Figure S3. Predicted partial secondary structure of the 5' UTR of simian HAV. Domains are labeled I to V. The putative initiator codon (AUG) and UUUCC sequence are indicated in red.


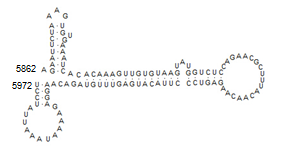


Figure S4. Predicted Cis-acting secondary structures of phopivirus in the 3D*^pol^*-coding region


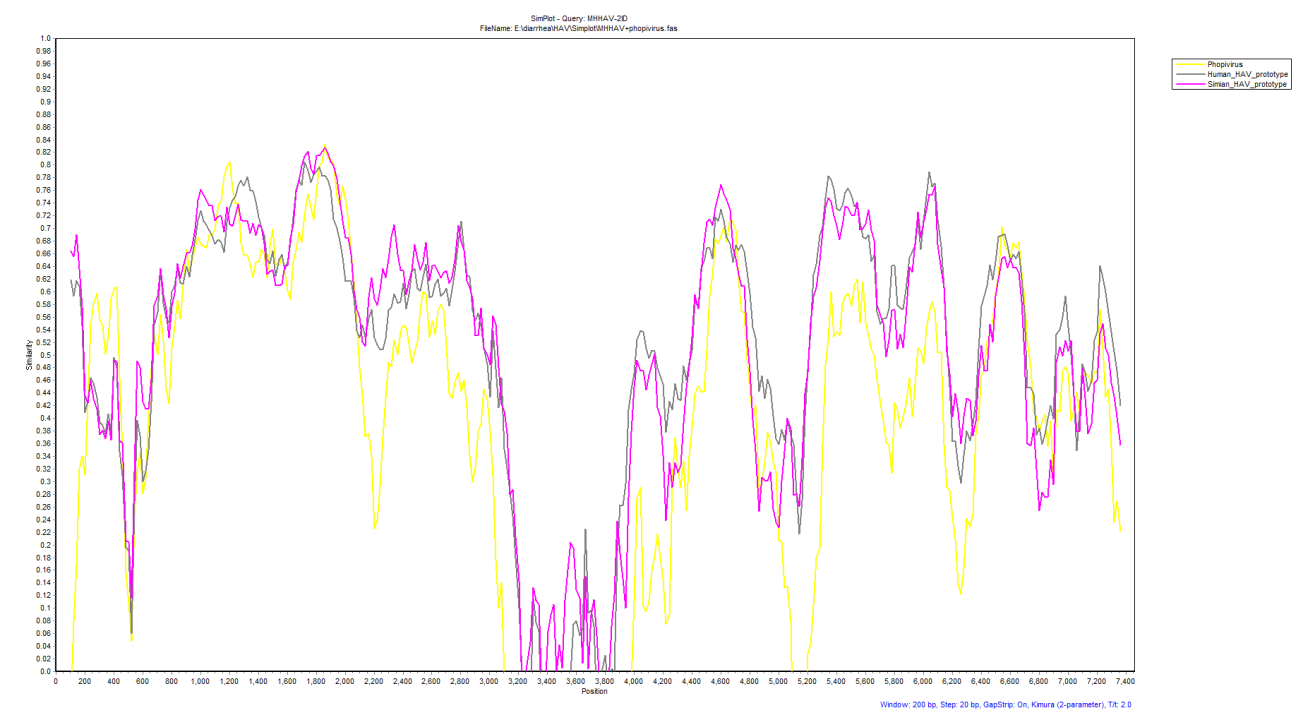


Figure S5. Recombination analysis of MHHAV with prototypic human and simian HAVs and phopivirus. The complete sequence of MHHAV was used as the query. No recombination was found upon comparison with the prototypic human and simian HAVs and phopivirus.
